# Results and Outcome Reporting In ClinicalTrials.gov, What Makes it Happen?

**DOI:** 10.1371/journal.pone.0037847

**Published:** 2012-06-13

**Authors:** Olga Kirillova

**Affiliations:** IICOLL LLC, Portland, Oregon, United States of America; University of Louisville, United States of America

## Abstract

**Background:**

At the end of the past century there were multiple concerns regarding lack of transparency in the conduct of clinical trials as well as some ethical and scientific issues affecting the trials’ design and reporting. In 2000 ClinicalTrials.gov data repository was developed and deployed to serve public and scientific communities with valid data on clinical trials. Later in order to increase deposited data completeness and transparency of medical research a set of restrains had been imposed making the results deposition compulsory for multiple cases.

**Methods:**

We investigated efficiency of the results deposition and outcome reporting as well as what factors make positive impact on providing information of interest and what makes it more difficult, whether efficiency depends on what kind of institution was a trial sponsor. Data from the ClinicalTrials.gov repository has been classified based on what kind of institution a trial sponsor was. The odds ratio was calculated for results and outcome reporting by different sponsors’ class.

**Results:**

As of 01/01/2012 118,602 clinical trials data deposits were made to the depository. They came from 9068 different sources. 35344 (29.8%) of them are assigned as FDA regulated and 25151 (21.2%) as Section 801 controlled substances. Despite multiple regulatory requirements, only about 35% of trials had clinical study results deposited, the maximum 55.56% of trials with the results, was observed for trials completed in 2008.

**Conclusions:**

The most positive impact on depositing results, the imposed restrains made for hospitals and clinics. Health care companies showed much higher efficiency than other investigated classes both in higher fraction of trials with results and in providing at least one outcome for their trials. They also more often than others deposit results when it is not strictly required, particularly, in the case of non-interventional studies.

## Introduction

Clinical studies are important and one of the biggest part of modern health care research in US. Besides they are ones of the most expensive and, dealing with human subject and people health, required to be done with a special care. At the end of the past century there were multiple concerns regarding lack of transparency in the conduct of clinical trials as well as some ethical and scientific issues affecting the trials’ design and reporting [1], [2]. In response on request to increase transparency of medical research and novel drugs development, the Food and Drug Administration issued a Modernization Act, Section 113 of which required the development of a data registry [3]. So, in February 2000 ClinicalTrials.gov data repository was developed and deployed (*Zarin, 2010 Everything You Ever Wanted to Know About ClinicalTrials.gov, on-line presentation*). At that time it was designed to help potential participants find trials, and was primarily focused on people with serious or life-threatening conditions. Since then through careful review process it was substantially improved to become more complete and accurate. In September 2007 Food and Drug Administration Amendments Act (FDAAA) was enacted with a legal requirement of trials registration for a broader group of trials than had previously been required under FDAMA [4]. In 2008, a database for reporting summary results was added to the registry [5]. Today technological advancement in large scale data processing, internet speed and cheap and getting cheaper electronic storage devices gives us an opportunity to deal with large scale data obtained from multiple sources and get a bigger picture of a clinical study.

In recent years there were several papers related to clinical trials: general reviews of clinical data repository ClinicalTrials.gov progress and development [5]–[7], investigation on how likely and soon a trial registered with ClinicalTrials.gov will result in a peer reviewed publication [8], [9], concerns related to completeness of an outcome in the trials reporting [10], and rigorous study of comparative effectiveness and its relationship to funding sources [11].

Characteristic feature of the previous research is that one or other kind of selection has been performed rather than meta-analysis of all data available. Another point with lack of attention, in our opinion, is classification of institutions sponsoring/conducting a trial.

In this study we performed overall meta-analysis of the clinical trials deposited into ClinicalTrials.gov repository as of January 1, 2012; developed advanced classification of trials sponsors and compare the results for different classes in two most important aspects of the deposited information: outcome reporting and deposition of clinical results data. Also we tried to decipher what factors make the results and outcome reporting more plausible or more difficult and whether it depends on the sponsor.

## Methods

### Data

Now significant number of clinical study records got public and everybody can download them from the site in a well structured format that makes the data processing easier and allows to keep the original structure and reduce potential errors usually occurring when plain text data need to be processed. We took the opportunity downloaded, processed and analyzed the data trying to decipher interesting regularities and to gain insight into the state of clinical research.

Data has been obtained from ClinicalTrials. gov repository. The last update has been done on 01/01/2012 and should contain all the clinical trials records as of the pointed date. The data were downloaded and imported into an in-house database. They were obtained in XML format, so all preexisting formatting has been saved. Parsing has been done by in-house developed *perl* script utilizing XML::Simple library for ease of XML parsing.

### Enhancement and Information Retrieval

**Figure 1:**
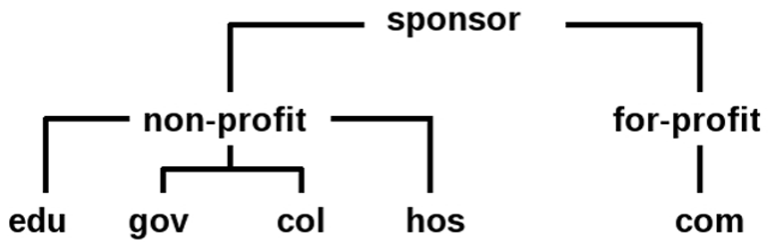
Schema of the classification.

While different kind of institutions take part in clinical research, they can be one of two types: for- or non-profit. Moreover, non-profit institutes are far non homogeneous among themself, they can have fairly different goals, primary duties, and follow different kind of regulations. So, in relation to a clinical trial the difference between a national institute and a hospital may be as big as between a university and a pharmaceutical company. Therefore, in the presented study non-profits have been further subdivided into four classes: Research/Educational Institutions (**edu**) consisting of universities, colleges, academia, and other alike institutes primarily focused on research and education; Hospitals & clinics (**hos**) - organizations with primary focus on providing health care service for people with health issues; collaborations including associations, networks and other non-government institutions able to include in itself different kind of participants (**col**) and national and government organizations (**gov**). For-profit sponsors were put into one class (**com**), including itself pharmaceutical and other commercial companies of health care sector conducted and deposited trials’ data. Classification schema is shown in Fig. 1. One has to note that the original data had sponsors classification. Namely, original classification had four classes: ‘Industry’, ‘NIH’, ‘Other’, and ‘U.S. Fed.’ We enhanced and slightly altered it in the way that ‘NIH’ and ‘U.S. Fed’ classes were joined into one class (**gov**). This class was extended to include other non US national and governments sponsored institutions. (**com**) class is quite consistent with ‘Industry’ in the original classification. And ‘Other’ has been distributed primarily into **col**, **hos** and **edu** classes.

Classification has been performed by in house text-mining classificator designed as:

1. define keywords for a given class (like ‘University’,’College’, ‘Università’, etc. for **edu** class; ‘Hospital’, ‘Clinics’, ‘Hôpitaux’, ‘Klinik’, etc. for **hos** class; ‘Company’, ‘Inc.’, ‘Corp.’, etc. for companies);
2. make dictionaries for each class;
3. define priorities, like ‘Hospital’ has higher priority than ‘University’ or ‘College’ in other words ‘University Hospital’ will be classified as **hos** rather than **edu.**

We passed all records through the classificator, with supplementary classification of records, which did not passed through, using agency class information from original classification of the sponsors. We used a leading sponsor of the trial in the classification. Then partial manual inspection and corrections were made.

So, we got trials distribution into classes as shown in Table 1.

**Table 1:** Classification of trials’ sponsors.

Research/Educational Institutions (**edu**)	Universities, colleges, academia, research institutes	32295 trials (27.2%)
Companies (**com**)	pharmaceutical and other for-profit businesses ofhealth care sector	38018 trials (32.1%)
National and Government Organizations (**gov**)	federal, municipal, and other government kind ofsponsored non-profit organizations	19414 trials (16.4%)
Hospitals & Clinics (**hos**)	hospitals & clinics sponsoring clinical trials	17198 trials (14.5%)
Collaborations (**col**)	organizations involving different institutions	10011 trials (8.4%)

Brief description and absolute and relative number of trials deposited into ClinicalTrials.gov 01/01/2012.

Overall correspondence between the depository classification and one described in this paper is shown in Table 2.

**Table 2:** Correspondence between classification described in this paper and one present in the ClinicalTrials.gov repository.

class (current)	class (original)	number of trials
com	Industry	37076
	Other	942
edu	Other	32118
	Industry	177
gov	U.S. Fed	1974
	NIH	9197
	Industry	776
	Other	7467
col	Other	9851
	Industry	160
hos	Other	17198
unclassified	Other	1666

One has to note, that it is very tricky to make a precise classification for over 118,000 trials coming from over 9,000 different sources, especially taking into account that deposits have been made from different countries and therefore, the sponsors are pointed in different languages. Besides, as it often happens, the texts may have multiple typographic errors. So, eventually our classification may have some errors but we do believe that it is not significant taking into account the set size. After the automatic classification manual refinement of the results has been made.

### Statistical Analysis

Since 1951 medical statisticians use the odds ratio (OR) as a measure of effect size, to describe the strength of association or non-independence between two binary data characteristics [12]. It is used as a descriptive statistic, where results are rather qualitative than quantitative or an answer on a question is either ‘yes’ or ‘no’. That perfectly suites our research of reporting clinical trials results and outcomes (for each trial one either has been reported or not). Additional beneficial feature of the odds ratio for our study is that it can be estimated using some types of non-random samples. The trials in the depository are definitely non-random taking into account that one sponsor usually deposits more than one trial.

So, we performed the odds ratio calculation as
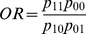
where *p_yx_* comes from the joint distribution of two binary random variables *X* and *Y*



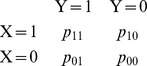
in our case:

X  = 1 if results were deposited (outcome reported), 0 otherwise,

Y  = 1 if the trial has been classified as belonging to a given class (edu, com, gov, hos), 0 otherwise.

We made conference interval estimate utilizing R software package (www.r-project.org), using *t*-test distribution and 95% confidence level.

## Results and Discussion

As of 01/01/2012 118,602 clinical trials data deposits were made to the depository. They came from 9068 different sources. 35344 (29.8%) of them are assigned as FDA regulated and 25151 (21.2%) as Section 801 controlled substances. 70929 (60%) trials had a treatment purpose.

**Figure 2:**
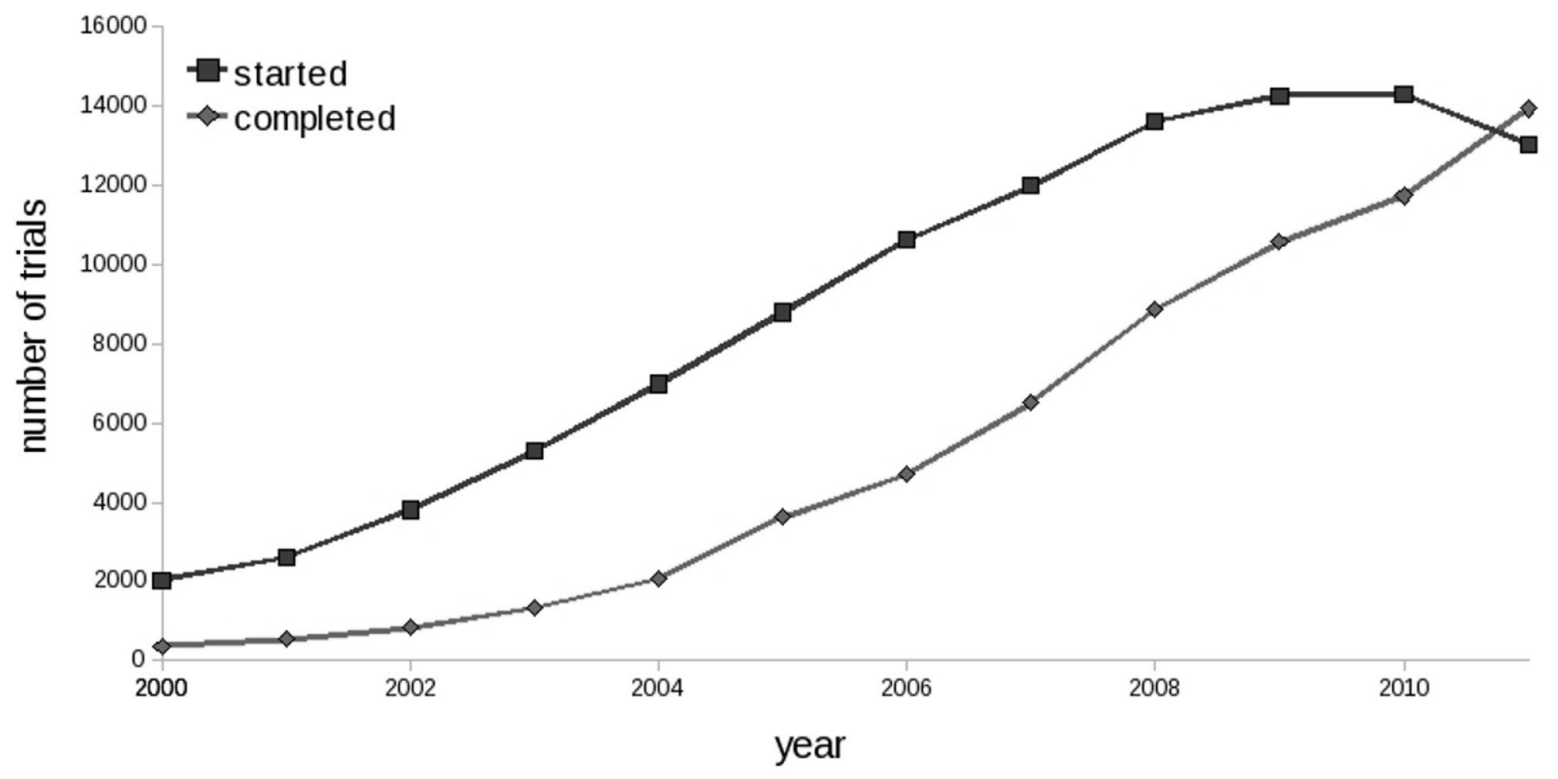
Number of trials started and completed each year since launching ClinicalTrials.gov repository.

To get a bigger picture, we calculated how number of started and completed trials progresses year over year from the lunch of the depository. 2011 was the only year through the decade of the repository existence when the number of trials completed exceeded the number of trials started (Fig. 2). In 2009 number of trials started came to some kind of saturation. Interestingly, it happened after the last recession (12/2007–6/2009) and the recession itself did not made a notable impact on clinical trials research (*US Business Cycle Expansions and Contractions*, http://www.nber.org/cycles.html).

**Figure 3:**
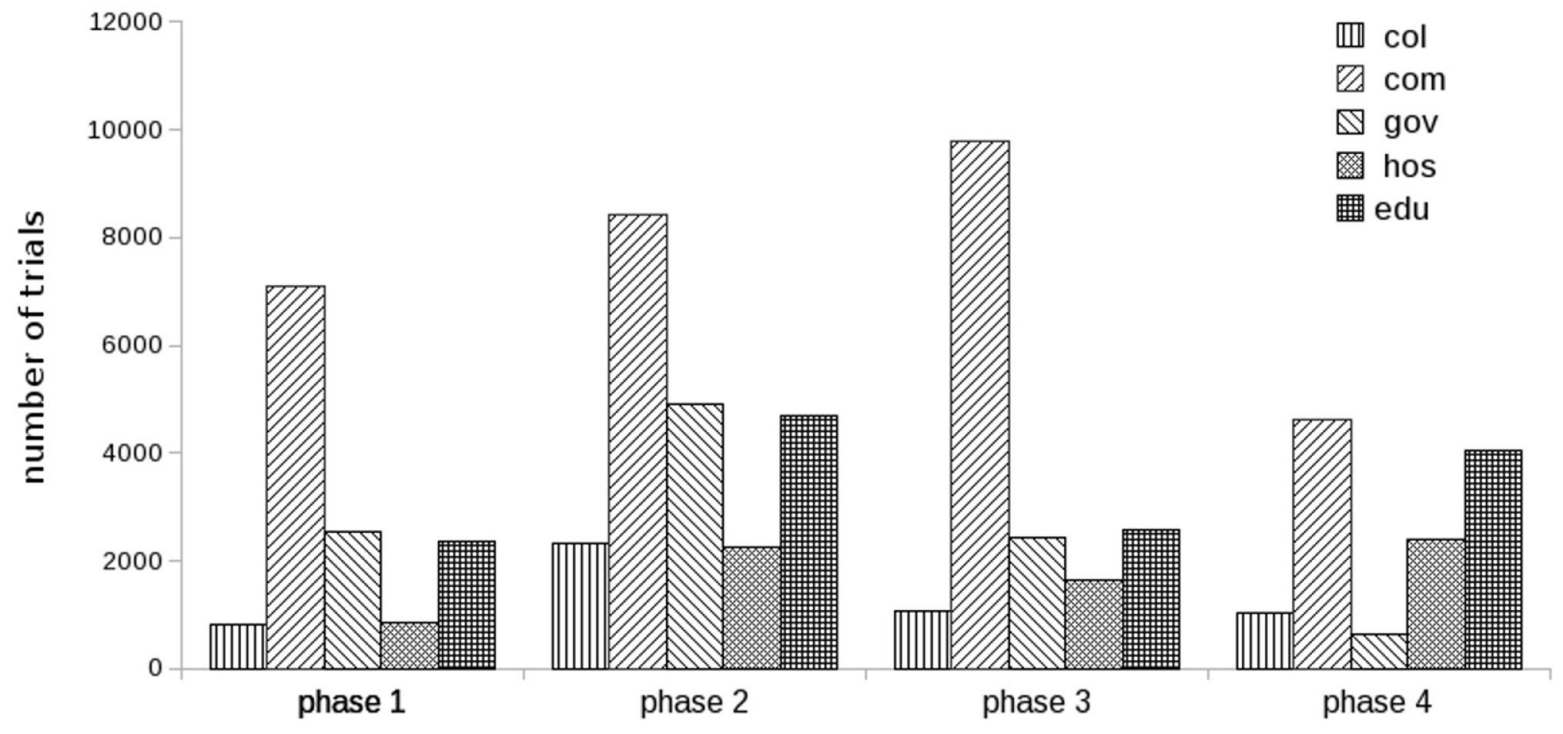
Number of trials assigned to different phases.

Another interesting feature we have observed, came from the distribution of trials among phases (1–4) for investigated classes (Fig. 3). For companies the number of trials per phase increases to phase 3, then it drops, **gov** and **col** classes have maximum at phase 2, while educational/research institutions have more trials for phase 4 than for phase 3. Currently we do not have an explanation for this phenomenon but would like to present it for community discussion.

### The Results and Outcome Reporting

In order to better understand drug safety and efficacy, biomedical community has to have clinical trials results not just a brief description. They also very important for establishing effectiveness measures “doing the right trials” [13]. So, availability of clinical results to public became one of the biggest concerns in clinical research [1], [5]. Besides, recently investigators have found that reporting, even among registered trials, was done selectively [14]. In response to these concerns, since 2007 FDAAA regulation requires to deposit the study results in case “all of the drugs, biologics, or devices used in that study have been approved by the FDA for at least one use” [4]. At the same time, the use of such registries as ClinicalTrials.gov has been demanded by the International Committee of Medical Journal Editors (ICMJE). As of 2005 the ICMJE has required trial registration before participant enrollment as a prerequisite for publication in any of its member journals [15].

Taking into account described above concerns as well as multiple efforts taken in recent years to achieve research transparency, spread from the FDA requirements to scientific publications in peer reviewed journals [16], we investigated how many trials have the results uploaded into the result database and what factors or regulations were more stimulating than others. Summary statistics for the deposits year-by-year, obeying different imposed requirements is given in Tables 3,4.

**Table 3:** Number of completed trials obeying imposed requirements with results and total, deposited into ClinicalTrials.gov.

completion year	Overall			FDA regulated			Section 801		
	with results	total	%	with results	total	%	with results	total	%
2011	169	13945	1.21	114	4475	2.55	93	3134	2.97
2010	894	11732	7.62	593	3899	15.21	491	2649	18.54
2009	1270	10588	11.99	899	3795	23.69	750	2643	28.38
2008	1328	8869	14.97	959	3084	31.1	814	2244	36.27
2007	385	6515	5.91	253	1464	17.28	190	990	19.19
2006	135	4714	2.86	99	848	11.67	56	523	10.71
2005	81	3632	2.23	61	657	9.28	32	408	7.84
2004	103	2076	4.96	90	530	16.98	31	333	9.31
2003	55	1337	4.11	52	389	13.37	16	248	6.45
2002	40	840	4.76	39	179	21.79	6	94	6.38
2001	16	547	2.93	16	84	19.05	9	47	19.15
2000 and before	20	1142	1.75	18	149	12.08	17	82	20.73
**total**	**4496**	**65937**	**6.82**	**3193**	**19553**	**16.33**	**2505**	**13395**	**18.7**

**Table 4:** Number of completed trials obeying imposed requirements with results and total, deposited into ClinicalTrials.gov.

completion year	phases 2–4			with publications			interventional			all requirements together		
	with results	total	%	with results	total	%	with results	total	%	with results	total	%
2011	113	6200	1.82	16	495	3.23	156	11194	1.39	6	61	9.84
2010	638	5445	11.72	71	602	11.79	785	9440	8.32	24	84	28.57
2009	973	5316	18.3	96	659	14.57	1188	8811	13.48	47	111	42.34
2008	1079	4733	22.8	138	710	19.44	1262	7396	17.06	75	135	55.56
2007	306	3815	8.02	57	637	8.95	373	5610	6.65	26	85	30.59
2006	94	2795	3.36	27	454	5.95	131	4062	3.23	16	45	35.56
2005	47	2268	2.07	12	396	3.03	76	3181	2.39	9	46	19.57
2004	46	1323	3.48	15	255	5.88	103	1858	5.54	7	27	25.93
2003	21	824	2.55	5	138	3.62	53	1176	4.51	2	17	11.76
2002	6	434	1.38	3	95	3.16	40	689	5.81	1	5	20
2001	8	291	2.75	1	67	1.49	16	429	3.73	1	4	25
2000 and before	18	485	3.71	6	167	3.59	20	698	2.87	6	14	42.86
**total**	**3349**	**33929**	**9.87**	**447**	**4675**	**9.56**	**4203**	**54544**	**7.71**	**220**	**634**	**34.7**

Overall, only 4927 (4%) of the deposits had reported clinical results and 6.82% of completed trials (having completion date as of 12/31/2011 or earlier). Certainly cumulative effect of taking into account all the imposed requirements as:

- a trial has to be completed as assigned in its overall status;
- FDA and specifically Section 801 regulations;
- availability of references to a peer reviewed journal (particularly ICMJE members);
- explicit notice of the phase (from 2 to 4);
- description of the study type as ‘interventional’

gives better chance for scientific community and general public to see the results but it still does not seems to be enough. Overall the cumulative requirements returned only about 35% of trials with the deposited results with the maximum 55.56% for trials completed in 2008. That means 3 years ago from the dates of the current analysis, while according to the FDA regulations the results have to be reported within 12 months of the completion date as it is specified in the filings. Section 801 of FDAAA requiring mandatory disclosure of specific clinical trial information on ClinicalTrials.gov, containing provisions for proof of compliance and authorizing penalties for noncompliance [4], alone has the highest impact on the results depositing. At the same time we note that 4701 trials do not obey any of the investigated requirements, set for the results deposition (or eventually it is not pointed explicitly in the filings) but trials’ conductors/sponsors deposited the results anyway.

The next point of our research was to check whether the trials data are different for different responsible institutions (sponsors). We look for how deposition of the results varies among different classes of sponsoring the trials institutions, taking into account all the applied regulations. It appears, government backed organizations less than others comply with the policy to deposit results of clinical trials. Industrial companies demonstrated the best performance in this aspect. And that would be expected taking into account that they have higher fraction of new drug applications and, therefore, more trials obeying restrictions imposed by the FDA regulations. Detailed statistics is present in Table 5.

**Table 5:** Number of trials with results and overall obeying all the imposed restrains as of 01/01/2012 for each assigned class.

class	trials with results	total	%
hos	21	65	32.31
edu	19	194	9.79
col	8	86	9.30
com	168	428	39.25
gov	10	156	6.41

Also clinical trials design and reporting policy requires investigators to disclosure outcomes of the conducted trials. This has well grounded reasons, at first, trial participants have the right to know abut known (from the previous study) risk by participating in trials. Secondly, public availability of this information will benefit next generation of clinical researchers and provides more rational use of healthcare resources. Eventually, outcome reporting may be biased, moreover, some researchers state that the bias occurs regardless of the funding source [17], [18], others claim that pharmaceutical industry companies are more prone to the bias [8], [19], [20]. Namely, the previous research showed that trials’ conductors are more enthusiastic for positive outcome reporting in literature [8]. Two aspects make this very likely: firstly, a paper with no results to show or describing something that did not went as expected, may be rejected in the review process, secondly, for companies there is no point to publish a negative outcome, since there is no peer reviewed publications in FDA requirements and a publication for them has rather an advertisement purpose. But depositing results and describing outcome in the repository gives community better chances to see how the trial has been conducted in detail and definitely is not so time and efforts consuming as writing a full paper. How different investigated classes use this opportunity?

4 of 5 assigned classes have very similar outcome reporting statistics close to 3/4 of deposits, while government class provides outcome description significantly more seldom than others. Educational/research class provides more comprehensive outcome description reporting more often not only the primary one but the secondary as well. Overall statistics for outcome reporting is considerably more optimistic than one for the results data being submitted into the repository. See Table 6 for details.

**Table 6:** Outcome reporting by different classes.

class	number oftrials withat least oneoutcome	%	number oftrials withmore thanone outcome	%
col	7288	72.8	2397	23.94
com	29433	77.42	10375	27.29
gov	7342	37.82	2182	11.24
hos	13197	76.74	4763	27.7
edu	24613	76.21	9758	30.22

**Table 7:** Odds ratios and confidence intervals for four investigated classes.

	results		outcome	
	OR	CI	OR	CI
com	7.7789	(7.7779, 7.7799)	1.8245	(1.8220, 1.8269)
gov	0.1320	(0.1318, 0.1322)	0.1995	(0.1982, 0.2009)
hos	0.3983	(0.3980, 0.3986)	1.5609	(1.5591, 1.5627)
edu	0.3240	(0.3237, 0.3244)	1.6123	(1.6100, 1.6146)

All trials were taken into account.

**Table 8:** Odds ratios and confidence intervals for four investigated classes.

	results		outcome	
	OR	CI	OR	CI
com	3.66	(3.62, 3.7)	1.4	(1.36, 1.44)
gov	0.16	(0.15, 0.17)	0.45	(0.43, 0.48)
hos	1.59	(1.58, 1.61)	1.28	(1.26, 1.3)
edu	0.43	(0.42, 0.45)	1.76	(1.74, 1.79)

For trials possessing the results deposition restrains.

### Odds Ratio

Switching from the data already known to an estimate of a future efficiency in the results and outcome reporting we utilized the odds ratio. Conceptually the odds of a successful event are defined as the ratio of the probability of success over the probability of failure. In our case OR allows us to estimate reporting efficiency as the ratio of cases where the results or outcome have been submitted into the depository (success) over cases where this has not been done and compare classes of the suggested classification to see whether the behavior is different depending on what kind of institution is responsible for a conducted trial. Since here we focus on the interclass difference, we omitted **col** class because of it intersects with others.

At first, we performed OR calculation for the entire set of trials. Here one can see substantial difference between **com** class and others in comparison of the results’ presence in the deposits (Table 7). Also one has to note that for the government sponsored class the OR is almost an order less than for others in outcome reporting. While, others are fairly close to each other. In other words, generally if a clinical trial has been conducted by a for-profit company, we have a higher chance to get the study results and outcome reported while the non-profit sector still needs substantial improvement especially regarding results of its trials. In this aspect our analysis does not support the previous research where the researchers concluded that for trials funded by industry, results reporting is less likely [21]. **edu** and **hos** classes are fairly close in both outcome and results reporting.

Then we look for how the numbers change if we take into account all mentioned above requirements, enforcing clinical results data deposition. In this case the investigated pool shrank to 584 trials. Calculating OR for this reduced set one can see some changes as for results as for the outcome reporting (Table 8).

Actually, the most positive impact on outcome reporting the imposed restrains made for hospitals and clinics. For companies both ratios got less than in no restrain case. Also now one can see considerable difference in effectiveness of results and outcome reporting for **edu** and **hos** classes. The restrains being developed for the results deposition, somehow made positive impact on outcome reporting for **edu** and **gov** classes. So, imposing restrains lead to results reporting efficiency decrease for **com**, increase substantially for **hos**, not significantly for **edu** and even less for **gov** classes.

### Interventions

Another characteristics impacting the reporting efficiency is what kind of intervention (if any) had been performed in the trial. Overall, top 3 intervention kinds are: drug, procedure and device. While all investigated classes have higher interest in new drug development. Companies are especially focused on drugs trials (73% of interventional trials) and pay surprisingly little attention to procedure development. For procedures the biggest contribution comes from hospitals (Table 9). One of possible explanation, coming from the data analysis, ‘procedure’ trials are often more time consuming than other. Namely, average duration of a ‘drug’ trial deposited into the repository is about 984 days, while for a ‘procedure’ trial it is 1302 days and a ‘device’ trial in average lasts for 1048 days. We compared efficiency for different classes and intervention types. Here efficiency is defined as percentage of number of trials with results for given conditions (class, intervention type) to the total number of trials for these conditions.

**Table 9:** Number of trials and results reporting efficiency for three most popular intervention types.

class	Drug		Procedure		Device	
	trials	efficiency	trials	efficiency	trials	efficiency
col	2014 (48.85%)	0.65%	496 (12.03%)	0.20%	402 (9.75%)	1.74%
com	12156 (73.18%)	4.70%	349 (2.1%)	3.72%	2099 (12.64%)	5.91%
gov	3397 (53.99%)	0.62%	692 (11%)	0.14%	282 (4.48%)	n/a
hos	2936 (39.17%)	1.77%	1498 (19.98%)	1.00%	848 (11.31%)	0.59%
edu	5739 (41.15%)	1.50%	2034 (14.58%)	0.98%	1307 (9.37%)	1.30%

‘Procedure’ trials for all except **hos** classes have lower efficiency in results reporting. For **col** and **com** ‘device’ trials have the highest efficiency. For **hos**, **gov** and **edu** classes the highest efficiency was observed for ‘drug’ trials.

### Enrollment

Patient enrollment is one of the most important and time-consuming aspects in clinical trials conduct. The depository requires to provide information on how many arms has been in the study and how many participants has been or anticipated to be enrolled in the trial.

Looking through the decade of the data collection for how many participants have been enrolled in a trial and how many arms a trial had. Appear, the number of arms pretty much consistent and in average is about 2±1 for all investigated classes. Data regarding enrollment, seem more interesting. While ClinicalTrials.gov general policy requires “Upon study completion, change Type to Actual and update the enrollment” (*ClinicalTrials.gov Protocol Data Element Definitions* http://prsinfo.clinicaltrials.gov/definitions.html), number of participants enrolled in the trials varies very widely from 0 to 99999999. 255 completed trials have 0 enrollment, 205 (80.4%) of them are interventional studies. Neither of them had the results deposited but 66 (25.9%) of them reported outcome of the study. 3 completed trials had 99999999 enrollment. All of them were classified as observational and neither of them had results deposited or outcome reported. Considering only completed trials with the results, minimum enrollment became 1 and maximum enrollment became 2323608. So, the results deposition substantially reduces the enrollment range and adds confidence to the data. Providing the results allows other researchers to get an idea of how to accomplish higher enrollment into a trial. Particularly, in the trial NCT01236053 with the highest enrollment assigned (2323608 participants) it is stated: “Patients were not recruited for nor enrolled in this study. This study is a retrospective observational study. Data from medical records or insurance claims databases are anonymized and used to develop a patient cohort. All diagnoses and treatments are recorded in the course of routine medical practice”.

The biggest overall variation was observed for government sponsored sector. Hospitals, according to the presented data, have an order higher enrollment than companies. That would be expected taking into account hospitals’ primary mission. At the same time, companies enrollment twice as big as one of educational/research class (Table 10).

**Table 10:** Clinical trials enrollment for different classes.

class	trials	max	average	participants total
col	4495	2120000	1308.19	5880327
com	25873	4300000	1055.88	27318851
gov	9550	99999999	33298.38	317999544
hos	7410	67128927	9493.47	70346597
edu	15111	10050956	1812.4	27387147
with reported outcome				
col	3295	2120000	1348.35	4442817
com	20634	4300000	801.89	16546258
gov	3633	200000	616.54	2239879
hos	5726	120000	287.34	1645327
edu	11583	2100000	1148.83	13306867
with clinical results deposited				
col	105	59510	866.72	91006
com	3482	2323608	1553.2	5408229
gov	118	4241	276.21	32593
hos	248	3362	147.96	36694
edu	463	59696	416.86	193007
plus interventional study				
col	97	6830	193	18744
com	3251	69274	478	1553678
gov	111	4241	268	29782
hos	235	1864	137	32195
edu	424	59696	417	176792

Overall status is ‘Completed’ or ‘Active, not recruiting’.

As we mentioned above the dispersion in the participants enrollment will be significantly decreased if we will consider only trials with reported outcome or submitted clinical results data. But impact of these two restrains is not the same: somehow companies have higher average enrollment for reported trial results than for the outcome, while four other classes have considerably lower all the numbers for trials with reported results. It would be expected to have higher enrollment for observational rather than interventional studies but somehow this impact is noted only for collaborations and companies, comparatively to trials with reported results. Also companies have more non-interventional studies with reported results.

Though there is no statistical correlation between enrollment and availability of deposited results, empirically, the bigger assigned in the trial enrollment the less chance to have reported results and/or an outcome.

More results of the meta-analysis are available at http://iicoll.com/Analytics/clinical_trials_report_2012.html.

### Conclusion

We investigated efficiency of results data deposition and outcome reporting in ClinicalTrials.gov repository. Also we researched what factors make positive impact on providing information of interest and what makes it more difficult, as well as whether this depends on what kind of institution is a sponsor of a trial.

While clinical results deposition is enforced up to the penalty by the FDA and more than encouraged by International Committee of Medical Journal Editors, overall the requirements making results deposition obligatory, returned only about 35% of trials with the deposited results, with the maximum 55.56% for trials completed in 2008.

Though multiple previous research pointed that the industry sector, corresponding **com** class in the current research, often has lower efficiency regarding presentation of their results in a literature [10], [11], [17]. Our study showed that completeness and efficiency of **com** class deposits into ClinicalTrials.gov repository seems to be much higher than for other classes in many aspects: higher fraction of trials with results both for overall and the restrained cases; the highest percentage in providing at least one outcome for the trials; significantly higher odds ratio for the results and slightly above others for outcom e (overall) depositing. Companies deposit their results even when it is not strictly required, particularly, they have more non-interventional studies with reported results. Industrial sector demonstrated the highest in average and total enrollment into its trails, confirmed by deposited result data.

The most positive impact on depositing results, the imposed restrains made for hospitals and clinics. Somehow the restrains also showed a positive influence on outcome reporting by educational and research institutions. For health care companies they did not seem to be an issue, moreover, both odds ratios got less than in no restrains case.
